# Supplementary material for: A shared speed encoding model for running and backing away behaviours in segregated neural circuits
Source: Nat Commun. 2026 Mar 17;17:4119. doi: 10.1038/s41467-026-70755-y (PMC13149553; doi:10.1038/s41467-026-70755-y)
Supplement: Supplementary file 1 — Supplementary Information [file 41467_2026_70755_MOESM1_ESM.pdf]

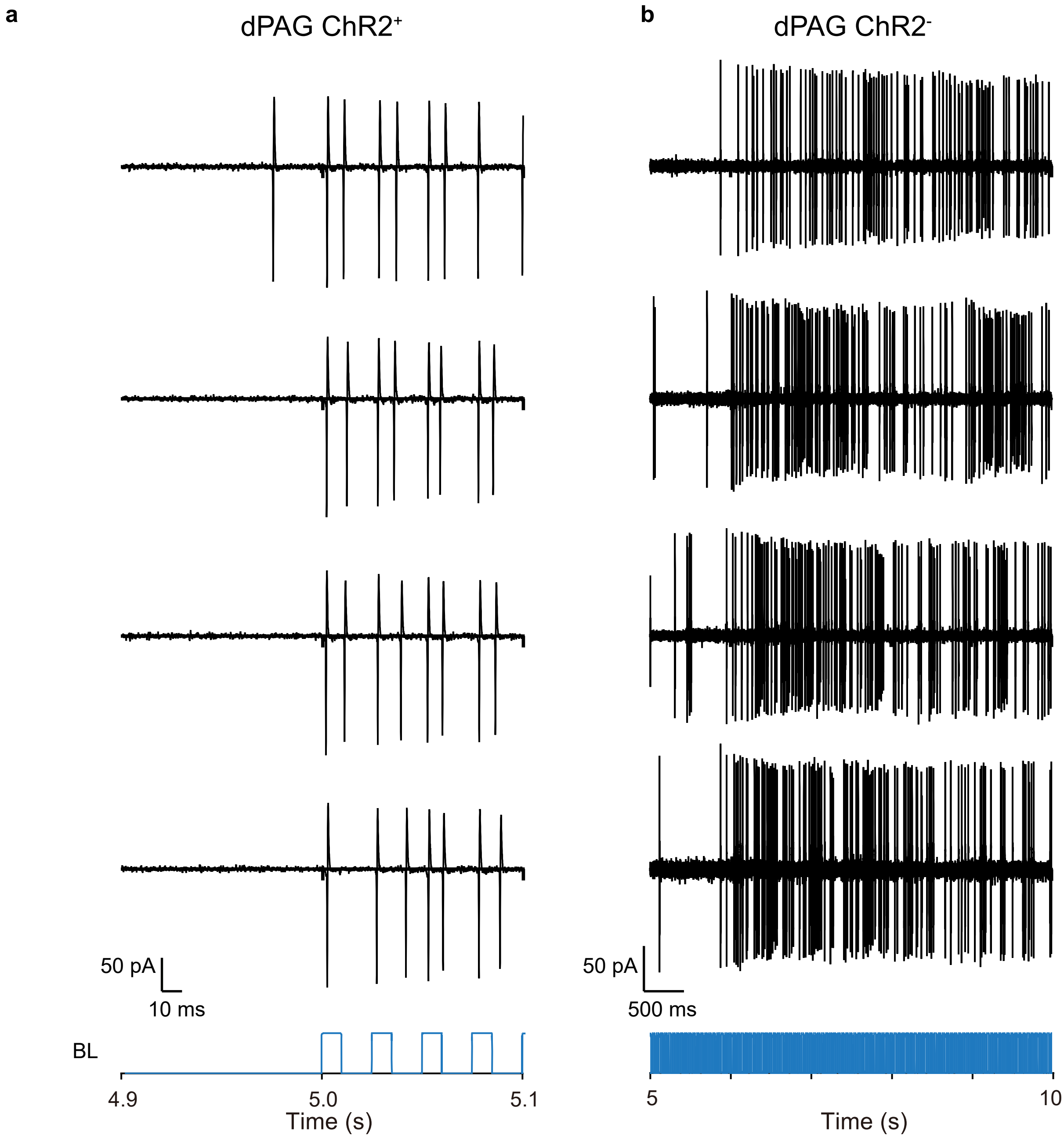

Supplementary Figure 1 | Optogenetic manipulation of dPAG neurons via direct activation versus indirect disynaptic inputs from TeA neurons.

a In vivo loose-patch recordings from dPAG neurons expressing ChR2 (ChR2+) (**Fig. 3e**).
b In vivo loose-patch recordings from dPAG neuron not expressing ChR2 (ChR2-) (TeA fibredPAG) (**Fig. 3b**). The duration of the light stimulation pulse was 10 ms. Source data are provided as a Source Data file.
